# Supplementary material for: Intracellular competition shapes plasmid population dynamics
Source: bioRxiv. 2025 Feb 20:2025.02.19.639193. Preprint. [Version 1] doi: 10.1101/2025.02.19.639193 (PMC11870584; doi:10.1101/2025.02.19.639193)
Supplement: Supplement 1 [file media-1.pdf]

Auxiliary plasmid:

GAGTTATACACAGGGCTGGGATCTATTCTTTTTATCTTTTTTTATTCTTTCTTTATTCTATAAATTATA
ACCACTTGAATATAAACAAAAAAAACACACAAAGGTCTAGCGGAATTTACAGAGGGTCTAGCAGAATTT
ACAAGTTTTCCAGCAAAGGTCTAGCAGAATTTACAGATACCCACAACTCAAAGGAAAAGGACTAGTAAT
TATCATTGACTAGCCCATCTCAATTGGTATAGTGATTAAAATCACCTAGACCAATTGAGATGTATGTCT
GAATTAGTTGTTTTCAAAGCAAATGAACTAGCGATTAGTCGCTATGACTTAACGGAGCATGAAACCAAG
CTAATTTTATGCTGTGTGGCACTACTCAACCCCACGATTGAAAACCCTACAAGGAAAGAACGGACGGTA
TCGTTCACTTATAACCAATACGCTCAGATGATGAACATCAGTAGGGAAAATGCTTATGGTGTATTAGCT
AAAGCAACCAGAGAGCTGATGACGAGAACTGTGGAAATCAGGAATCCTTTGGTTAAAGGCTTTGAGATT
TTCCAGTGGACAAACTATGCCAAGTTCTCAAGCGAAAAATTAGAATTAGTTTTTAGTGAAGAGATATTG
CCTTATCTTTTCCAGTTAAAAAAATTCATAAAATATAATCTGGAACATGTTAAGTCTTTTGAAAACAAA
TACTCTATGAGGATTTATGAGTGGTTATTAAAAGAACTAACACAAAAGAAAACTCACAAGGCAAATATA
GAGATTAGCCTTGATGAATTTAAGTTCATGTTAATGCTTGAAAATAACTACCATGAGTTTAAAAGGCTT
AACCAATGGGTTTTGAAACCAATAAGTAAAGATTTAAACACTTACAGCAATATGAAATTGGTGGTTGAT
AAGCGAGGCCGCCCGACTGATACGTTGATTTTCCAAGTTGAACTAGATAGACAAATGGATCTCGTAACC
GAACTTGAGAACAACCAGATAAAAATGAATGGTGACAAAATACCAACAACCATTACATCAGATTCCTAC
CTACATAACGGACTAAGAAAAACACTACACGATGCTTTAACTGCAAAAATTCAGCTCACCAGTTTTGAG
GCAAAATTTTTGAGTGACATGCAAAGTAAGTATGATCTCAATGGTTCGTTCTCATGGCTCACGCAAAAA
CAACGAACCACACTAGAGAACATACTGGCTAAATACGGAAGGATCTGAGGTTCTTATGGCTCTTGTATC
TATCAGTGAAGCATCAAGACTAACAAACAAAAGTAGAACAACTGTTCACCGTTACATATCAAAGGGAAA
ACTGTCCATATGCACAGATGAAAACGGTGTAAAAAAGATAGATACATCAGAGCTTTTACGAGTTTTTGG
TGCATTCAAAGCTGTTCACCATGAACAGATCGACAATGTAACAGATGAACAGCATGTAACACCTAATAG
AACAGGTGAAACCAGTAAAACAAAGCAACTAGAACATGAAATTGAACACCTGAGACAACTTGTTACAGC
TCAACAGTCACACATAGACAGCCTGAAACAGGCGATGCTGCTTATCGAATCAAAGCTGCCGACAACACG
GGAGCCAGTGACGCCTCCCGTGGGGAAAAAATCATGGCAATTCTGGAAGAAATAGCGCTTTCAGCCGGC
AAACCGGCTGAAGCCGGATCTGCGATTCTGATAACAAACTAGCAACACCAGAACAGCCCGTTTGCGGGC
AGCAAAACCCGTACCCTAGGTCTAGGGCGGCGGATTTGTCCTACTCAGGAGAGCGTTCACCGACAAACA
ACAGATAAAACGAAAGGCCCAGTCTTTCGACTGAGCCTTTCGTTTTATTTGATGCCTCTAGAGCTTGCA
TGCCTGCAGGTTAAATGCGGCGGCTAATATAGCTGCTCAGATAATCCAGCACTTCTTGCGCAATAATGC
CGTTCCACGCCGCATAGCGGGTGCTGCCGCCGGTGCTGCCTTTCAGCTGTTCAATATGGCGCCATTCTT
CAATCGGGTTGGTTTCATCTTTCCACGGAATCATTTCTTTGCTAATCTGATCATAGCCATAATAGCCGC
TCACCAGCGCAAAATAATGATCCGGAATCGCGGTCACTTGATGGGTATAGGTGGTGCGCGCCACCACGC
TCGCGCGTTTATCGCTCCAGTTGCCCACCACGTTGGTCAGTTCGGTCAGGCTTTTCATGCTCAGAAAGC
TCGTCATCAGATGGCGGCCAATATGGCTTTTCGGGCCGTTTTTAATCGCCAGAATGCTATACGGCGCGT
TGCTTTTCAGCGCTTTGTTATAGCTGCGCACCAGGTTATCTTTCAGCAGCTGATACTGCTGTTTGTTGC
CGCTGCTGCTGCTCGTTTTGTTAATGCGTTTCGGCACCGGTTCGCTATAGCGCAGAAATTCATCCAGAT
ACACCAGGCTATCCAGGCGGCCTTTCGCGCTAAAAAAATAAATATGGCGGCTCACGCCGGTTTTGGTTT
CGGTCACCAGGCACTGAATAATCACGCCCAGATATTCGTTCTGAATCAGTTTAAAGCTCTGCGGATCCA
CGTTTTTAATATCGCTAAAGCGCGCGCAGTTCACAAAGGTCGCCAGAAACAGAAACTGATACTGCGCTT
TGGTTTTGGTATAGCGGCTCGTATATTCAAAGCTATCCAGAATTTTTTCCGCAATGTTCCACACGCTTT
CATCTTCGTTCAGCAGCGCTTTCAGCATTTTTTTGCTATGGCTGTTGCCTTTTTCCACTTCTTCCGGGC
TTTCAAACTGCAGCTGCAGGTTGCTCACAATATCGGTCACATCGCTCTGTTCTTTCTGACCATAATACG
GAATAATGGTAAATTCCCAGCCCGGGATCAGCTTCTGCAGGCTCGCTTGCAGAATCGCCGCTTTCTGCG
TTTTATATTTAAACTGCAGGGTTTTTTTCACCACATCATACTGCAGGCTTTTGCTAATAATGGTGTTAT

AGCTCAGAAAGGTCGCGCGTTTAATCGCCGCGCCGTTATGGGTAATCATCCAGCACAGATAGGTCAGTT
CCGCCGCGCAGCTCGCCAGTTTTTCGCCGCTCGGTTCGCCAAAGCGCGCAATAAACTGACTCACCAGCA
CTTTCGGCGGGGTTTTATACAGAATATCAAATTTGCTCATGGTGAATTCCTCCTGCTAGCCCAAAAAAA
CGGGTATGGAGAAACAGTAGAGAGTTGCGATAAAAAGCGTCAGGTAGGATCCGCTAATCTTATGGATAA
AAATGCTATGGCATAGCAAAGTGTGACGCCGTGCAAATAATCAATGTGGACTTTTCTGCCGTGATTATA
GACACTTTTGTTACGCGTTTTTGTCATGGCTTTGGTCCCGCTTTGTTACAGAATGCTTTTAATAAGCGG
GGTTACCGGTTTGGTTAGCGAGAAGAGCCAGTAAAAGACGCAGTGACGGCAATGTCTGATGCAATATGG
ACAATTGGTTTCTTCTCTGAATGGCGGGAGTATGAAAAGTATGGCTGAAGCGCAAAATGATCCCCTGCT
GCCGGGATACTCGTTTAATGCCCATCTGGTGGCGGGTTTAACGCCGATTGAGGCCAACGGTTATCTCGA
TTTTTTTATCGACCGACCGCTGGGAATGAAAGGTTATATTCTCAATCTCACCATTCGCGGTCAGGGGGT
GGTGAAAAATCAGGGACGAGAATTTGTTTGCCGACCGGGTGATATTTTGCTGTTCCCGCCAGGAGAGAT
TCATCACTACGGTCGTCATCCGGAGGCTCGCGAATGGTATCACCAGTGGGTTTACTTTCGTCCGCGCGC
CTACTGGCATGAATGGCTTAACTGGCCGTCAATATTTGCCAATACGGGGTTCTTTCGCCCGGATGAAGC
GCACCAGCCGCATTTCAGCGACCTGTTTGGGCAAATCATTAACGCCGGGCAAGGGGAAGGGCGCTATTC
GGAGCTGCTGGCGATAAATCTGCTTGAGCAATTGTTACTGCGGCGCATGGAAGCGATTAACGAGTCGCT
CCATCCACCGATGGATAATCGGGTACGCGAGGCTTGTCAGTACATCAGCGATCACCTGGCAGACAGCAA
TTTTGATATCGCCAGCGTCGCACAGCATGTTTGCTTGTCGCCGTCGCGTCTGTCACATCTTTTCCGCCA
GCAGTTAGGGATTAGCGTCTTAAGCTGGCGCGAGGACCAACGTATCAGCCAGGCGAAGCTGCTTTTGAG
CACCACCCGGATGCCTATCGCCACCGTCGGTCGCAATGTTGGTTTTGACGATCAACTCTATTTCTCGCG
GGTATTTAAAAAATGCACCGGGGCCAGCCCGAGCGAGTTCCGTGCCGGTTGTGAAGAAAAAGTGAATGA
TGTAGCCGTCAAGTTGTCATAATTGGTAACGAATCAGACAATTGACGGCTTGACGGAGTAGCATAGGGT
TTGCAGAATCCCTGCTTCGTCCATTTGACAGGCACATTATGCATGCCCGTAAAGTTATCCAGCAACCAC
TCATAGACCTAGGGCAGCAGATAGGGACGACGTGGTGTTAGCTGTGGTGAAGACGAAAGGGCCTCGTGA
TACGCCTATTTTTATAGGTTAATGTCATGATAATAATGGTTTCTTAGACGTCGGAATTGCCAGCTGGTT
AATTAAGGTTTCTTAGACGTCAGGTGGCACTTTGACTGGAGTTCAGACGTGTGCTCTTCCGATCTGTGG
GTACCGCGCCCTCTGGTAAGTGATCGGCACGTAAGAGGTTCCAACTTTCACCATAATGAAATAAGATCA
CTACCGGGCGTATTTTTTGAGTTGTCGAGATTTTCAGGAGCTAAGGAAGCTAAAATGGAGAAAAAAATC
ACTGGATATACCACCGTTGATATATCCCAATGGCATCGTAAAGAACATTTTGAGGCATTTCAGTCAGTT
GCTCAATGTACCTATAACCAGACCGTTCAGCTGGATATTACGGCCTTTTTAAAGACCGTAAAGAAAAAT
AAGCACAAGTTTTATCCGGCCTTTATTCACATTCTTGCCCGCCTGATGAATGCTCATCCGGAATTACGT
ATGGCAATGAAAGACGGTGAGCTGGTGATATGGGATAGTGTTCACCCTTGTTACACCGTTTTCCATGAG
CAAACTGAAACGTTTTCATCGCTCTGGAGTGAATACCACGACGATTTCCGGCAGTTTCTACACATATAT
TCGCAAGATGTGGCGTGTTACGGTGAAAACCTGGCCTATTTCCCTAAAGGGTTTATTGAGAATATGTTT
TTCGTCTCAGCCAATCCCTGGGTGAGTTTCACCAGTTTTGATTTAAACGTGGCCAATATGGACAACTTC
TTCGCCCCCGTTTTCACCATGGGCAAATATTATACGCAAGGCGACAAGGTGCTGATGCCGCTGGCGATT
CAGGTTCATCATGCCGTTTGTGATGGCTTCCATGTCGGCAGAATGCTTAATGAATTACAACAGTACTGC
GATGAGTGGCAGGGCGGGGCGTAATAAGGACTCTGGGATCTCTGCAGTCGCGATGATTAATTAATTCAG
AACGCTCGGTTGCCGCCGGGCGTTTTTTATGCATGAGAATCCTGGCGGGTCTGTCGACGCACTAGGGAC
AGTAAGACGGGTAAGCCTGTTGATGATACCGCTGCCTTACTGGGTGCATTAGCCAGTCTGAATGACCTG
TCACGGGATAATCCGAAGTGGTCAGACTGGAAAATCAGAGGGCAGGAACTGCTGAACAGCAAAAAGTCA
GATAGCACCACATAGCAGACCCGCCATAAAACGCCCTGAGAAGCCCGTGACGGGCTTTTCTTGTATTAT
GGGTAGTTTCCTTGCATGAATCCATAAAAGGCGCCTGTAGTGCCATTTACCCCCATTCACTGCCAGAGC
CGTGAGCGCAGCGAACTGAATGTCACGAAAAAGACAGCGACTCAGGTGCCTGATGGTCGGAGACAAAAG
GAATATTCAGCGATTTGCCCGAGCTTGCGAGGGTGCTACTTAAGCCTTTAGGGTTTTAAGGTCTGTTTT
GTAGAGGAGCAAACAGCGTTTGCGACATCCTTTTGTAATACTGCGGAACTGACTAAAGTAGT

PSC101 origin NNNNN; Patagonian FLP NNNNN; Chloramphenicol resistance cassette NNNNN

Target plasmid monomer:

TTTCCATAGGCTCCGCCCCCCTGACGAGCATCACAAAAATCGACGCTCAAGTCAGAGGTGGCGAAACCC
GACAGGACTATAAAGATACCAGGCGTTTCCCCCTGGAAGCTCCCTCGTGCGCTCTCCTGTTCCGACCCT
GCCGCTTACCGGATACCTGTCCGCCTTTCTCCCTTCGGGAAGCGTGGCGCTTTCTCATAGCTCACGCTG
TAGGTATCTCAGTTCGGTGTAGGTCGTTCGCTCCAAGCTGGGCTGTGTGCACGAACCCCCCGTTCAGCC
CGACCGCTGCGCCTTATCCGGTAACTATCGTCTTGAGTCCAACCCGGTAAGACACGACTTATCGCCACT
GGCAGCAGCCACTGGTAACAGGATTAGCAGAGCGAGGTATGTAGGCGGTGCTACAGAGTTCTTGAAGTG
GTGGCCTAACTACGGCTACACTAGAAGGACAGTATTTGGTATCTGCGCTCTGCTGAAGCCAGTTACCTT
CGGAAAAAGAGTTGGTAGCTCTTGATCCGGCAAACAAACCACCGCTGGTAGCGGTGGTTTTTTTGTTTG
CAAGCAGCAGATTACGCGCAGAAAAAAAGGATCTCAAGAAGATCCTTTGATCTTTTCTACGGGGTCTGA
CGCTCAGTGGGTCGACGTCTTACTGTCCCTAGTGCTTGGATTCTCACCAATAAAAAACGCCCGGCGGCA
ACCGAGCGTTCTGAACAAATCCAGATGGAGTTCTGAGGTCATTACTGGATCTATCAACAGGAGTCCAAG
CGAGCTCTCGAACCCCAGAGTCCCGCTCAGAAGAACTCGTCAAGAAGGCGATAGAAGGCGATGCGCTGC
GAATCGGGAGCGGCGATACCGTAAAGCACGAGGAAGCGGTCAGCCCATTCGCCGCCAAGCTCTTCAGCA
ATATCACGGGTAGCCAACGCTATGTCCTGATAGCGGTCCGCCACACCCAGCCGGCCACAGTCGATGAAT
CCAGAAAAGCGGCCATTTTCCACCATGATATTCGGCAAGCAGGCATCGCCATGGGTCACGACGAGATCC
TCGCCGTCGGGCATGCGCGCCTTGAGCCTGGCGAACAGTTCGGCTGGCGCGAGCCCCTGATGCTCTTCG
TCCAGATCATCCTGATCGACAAGACCGGCTTCCATCCGAGTACGTGCTCGCTCGATGCGATGTTTCGCT
TGGTGGTCGAATGGGCAGGTAGCCGGATCAAGCGTATGCAGCCGCCGCATTGCATCAGCCATGATGGAT
ACTTTCTCGGCAGGAGCAAGGTGAGATGACAGGAGATCCTGCCCCGGCACTTCGCCCAATAGCAGCCAG
TCCCTTCCCGCTTCAGTGACAACGTCGAGCACAGCTGCGCAAGGAACGCCCGTCGTGGCCAGCCACGAT
AGCCGCGCTGCCTCGTCCTGCAGTTCATTCAGGGCACCGGACAGGTCGGTCTTGACAAAAAGAACCGGG
CGCCCCTGCGCTGACAGCCGGAACACGGCGGCATCAGAGCAGCCGATTGTCTGTTGTGCCCAGTCATAG
CCGAATAGCCTCTCCACCCAAGCGGCCGGAGAACCTGCGTGCAATCCATCTTGTTCAATCATGCGAAAC
GATCCTCATCCTGTCTCTTGATCAGATCTTGATCCCCTGCGCCATCAGATCCTTGGCGGCAAGAAAGCC
ATCCAGTTTACTTTGCAGGGCTTCCCAACCTTACCAGAGGGCGCCTGACTGGAGTTCAGACGTGTGCTC
TTCCGATCTGTGGGTACCTTGTTGACTAGTGTGAGATCGGAAGAGCGTCGTGTAGGGAAAGAGTGTCCA
GCTGGCAATTCCGTTAATTAACACCTGACGTCTAAGAAACCATTATTATCATGACATTAACCTATAAAA
ATAGGCGTATCACGAGGCCCTTTCGTCTTCAAGAAGTTCCTATTCTCTAGAAAGTATAGGAACTTCTCA
CCACAGCTAACACCACGTCGTCCCTATCTGCTGCCCTAGGTCTATGAGTGGTTGCTGGATAAC**~Promo
ter~**ATATTCAGGGAGACCACAACGGTTTCCCTCTACAAATAATTTTGTTTAACTTTTCTAGATTTAAG
AAGGAGATATACAT**~Insert~**TAAATGTCCAGACCTGCAGGCATGCAAGCTCTAGAGGCATCAAATAA
AACGAAAGGCTCAGTCGAAAGACTGGGCCTTTCGTTTTATCTGTTGAAATGCACCAAAAACTCGTAAAA
GCTCTGATGTATCTATCTTTTTTACACCGTTTTCATCTGTGCATATGGACAGTTTTCCCT**~Restrict
ionAssemblySite~**GCTAGCCTCGGGCAGCGTTGGGTCCTGGCCACGGGTGCGCATGATCGTGCTCC
TGTCGTTGAGGACCCGGCTAGGCTGGCGGGGTTGCCTTACTGGTTAGCAGAATGAATCACCGATACGCG
AGCGAACGTGAAGCGACTGCTGCTGCAAAACGTCTGCGACCTGAGCAACAACATGAATGGTCTTCGGTT
TCCGTGTTTCGTAAAGTCTGGAAACGCGGAAGTCAGCGCCCTGCACCATTATGTTCCGGATCTGCATCG
CAGGATGCTGCTGGCTACCCTGTGGAACACCTACATCTGTATTAACGAAGCGCTGGCATTGACCCTGAG
TGATTTTTCTCTGGTCCCGCCGCATCCATACCGCCAGTTGTTTACCCTCACAACGTTCCAGTAACCGGG
CATGTTCATCATCAGTAACCCGTATCGTGAGCATCCTCTCTCGTTTCATCGGTATCATTACCCCCATGA

ACAGAAATCCCCCTTACACGGAGGCATCAGTGACCAAACAGGAAAAAACCGCCCTTAACATGGCCCGCT
TTATCAGAAGCCAGACATTAACGCTTCTGGAGAAACTCAACGAGCTGGACGCGGATGAACAGGCAGACA
TCTGTGAATCGCTTCACGACCACGCTGATGAGCTTTACCGCAGCTGCCTCGCGCGTTTCGGTGATGACG
GTGAAAACCTCTGACACATGCAGCTCCCGGAGACGGTCACAGCTTGTCTGTAAGCGGATGCCGGGAGCA
GACAAGCCCGTCAGGGCGCGTCAGCGGGTGTTGGCGGGTGTCGGGGCGCAGCCATGACCCAGTCACGTA
GCGATAGCGGAGTGTATACTGGCTTAACTATGCGGCATCAGAGCAGATTGTACTGAGAGTGCACCATAT
GCGGTGTGAAATACCGCACAGATGCGTAAGGAGAAAATACCGCATCAGGCGCTCTTCCGCTTCCTCGCT
CACTGACTCGCTGCGCTCGGTCGTTCGGCTGCGGCGAGCGGTATCAGCTCACTCAAAGGCGGTAATACG
GTTATCCACAGAATCAGGGGATAACGCAGGAAAGAACATGTGAGCAAAAGGCCAGCAAAAGGCCAGGAA
CCGTAAAAAGGCCGCGTTGCTGGCGTT

PBR322 origin NNNNN; FRT site NNNNN; Kanamycin resistance cassette NNNNN; Barcode region NNNNN

Promoters:
ProA
TTTACGGGCATGCATAAGGCTCG**TAGGCT**
ProC
TTTACGGGCATGCATAAGGCTCG**TATGAT**

Restriction assembly site for mScarlet-I plasmid:
GGCGCGCCGAGAGG**GGATCC**
Restriction assembly site for mWatermelon plasmid:
GGATCCGAGAGG**GGCGCGCC**

Inserts:
mScarlet-I
ATGAGTAAAGGAGAAGCTGTTATTAAAGAGTTCATGCGCTTCAAAGTTCACATGGAGGGTTCTATGAAC
GGTCACGAGTTCGAGATCGAAGGCGAAGGCGAGGGCCGTCCGTATGAAGGCACCCAGACCGCCAAACTG
AAAGTGACTAAAGGCGGCCCGCTGCCTTTTTCCTGGGACATCCTGAGCCCGCAATTTATGTACGGTTCT
AGGGCGTTCATCAAACACCCAGCGGATATCCCGGACTATTATAAGCAGTCTTTTCCGGAAGGTTTCAAG
TGGGAACGCGTAATGAATTTTGAAGATGGTGGTGCCGTGACCGTCACTCAGGACACCTCCCTGGAGGAT
GGCACCCTGATCTATAAAGTTAAACTGCGTGGTACTAATTTTCCACCTGATGGCCCGGTGATGCAGAAA
AAGACGATGGGTTGGGAGGCGTCTACCGAACGCTTGTATCCGGAAGATGGTGTGCTGAAAGGCGACATT
AAAATGGCCCTGCGCCTGAAAGATGGCGGCCGCTATCTGGCTGACTTCAAAACCACGTACAAAGCCAAG
AAACCTGTGCAGATGCCTGGCGCGTACAATGTGGACCGCAAACTGGACATCACCTCTCATAATGAAGAT
TATACGGTGGTAGAGCAATATGAGCGCTCCGAGGGTCGTCATTCTACCGGTGGCATGGATGAACTATAC
AAA
mWatermelon
ATGAGTAAAGGAGAAGCTCTGATTAAAGAGTACATGCGCTTCAAAGTTCACATGGAGGGTTCTATGGAC
GGTCACGAGTTCGAGATCGAAGGCGAAGGCGAGGGCCGTCCGTATGAAGGCACCCATACCGCCAAACTG
AAAGTGACTAAAGGCGGCCCGCTGCCTTTTTCCTGGGACATCCTGAGCCCGCAATTTGGCTACGGTTCT
AGGGCGTTCATCAAACACCCAGCGGATATCCCGGACTATTATAAGCAGTCTTTTCCGGAAGGTTTCAAG
TGGGAACGCGTAATGAATTTTGAAGATGGTGGTGCCGTGACCGTCACTCAGGACACCTCCCTGGAGGAT
GGCACCCTGATCCATAAAGTTAAACTGCGTGGTACTAATTTTCCACCTGATGGCCCGGTGATGCAGCGT

AAGACGATGGGTTGGGAGGCGTCTACCGAACGCTTGTATCCGGAAGATGGTGTGCTGAAAGGCGACATT
AAAATGGCCCTGCGCCTGAAAGATGGCGGCCGCTATCTGGCTGACTGCAAAACCACGTACAAAGCCAAG
AAACCTGTGCAGATGCCTGGCGCGTACAATGTGGACCGCAAACTGGACATCACCTCTCATAATGAAGAT
TATACGGTGGTAGAGCAATATGAGCGCTCCGAGGGTCGTCATTCTACCGGTGGCATGGATGAACTATAC
AAA

mWatermelon-DfrA

ATGAGTAAAGGAGAAGCTCTGATTAAAGAGTACATGCGCTTCAAAGTTCACATGGAGGGTTCTATGGAC
GGTCACGAGTTCGAGATCGAAGGCGAAGGCGAGGGCCGTCCGTATGAAGGCACCCATACCGCCAAACTG
AAAGTGACTAAAGGCGGCCCGCTGCCTTTTTCCTGGGACATCCTGAGCCCGCAATTTGGCTACGGTTCT
AGGGCGTTCATCAAACACCCAGCGGATATCCCGGACTATTATAAGCAGTCTTTTCCGGAAGGTTTCAAG
TGGGAACGCGTAATGAATTTTGAAGATGGTGGTGCCGTGACCGTCACTCAGGACACCTCCCTGGAGGAT
GGCACCCTGATCCATAAAGTTAAACTGCGTGGTACTAATTTTCCACCTGATGGCCCGGTGATGCAGCGT
AAGACGATGGGTTGGGAGGCGTCTACCGAACGCTTGTATCCGGAAGATGGTGTGCTGAAAGGCGACATT
AAAATGGCCCTGCGCCTGAAAGATGGCGGCCGCTATCTGGCTGACTGCAAAACCACGTACAAAGCCAAG
AAACCTGTGCAGATGCCTGGCGCGTACAATGTGGACCGCAAACTGGACATCACCTCTCATAATGAAGAT
TATACGGTGGTAGAGCAATATGAGCGCTCCGAGGGTCGTCATTCTACCGGTGGCATGGATGAACTATAC
AAATAAATGTCCAGACCTGCAGGCAGGGTGCGGCGGGCACACCGCCTCCCCTGAGCTGTCACCGGATGT
GCTTTCCGGTCTGATGAGTCCGTGAGGACGAAACAGCCTCTACAAATAATTTTGTTTAA**~RBS~**ATGAA
ACTATCACTAATGGTAGCTATATCGAAGAATGGAGTTATCGGGAATGGCCCTGATATTCCATGGAGTGC
CAAAGGTGAACAGCTCCTGTTTAAAGCTATTACCTATAACCAATGGCTGTTGGTTGGACGCAAGACTTT
TGAATCAATGGGAGCATTACCCAACCGAAAGTATGCGGTCGTAACACGTTCAAGTTTTACATCTGACAA
TGAGAACGTATTGATCTTTCCATCAATTAAAGATGCTTTAACCAACCTAAAGAAAATAACGGATCATGT
CATTGTTTCAGGTGGTGGGGAGATATACAAAAGCCTGATCGATCAAGTAGATACACTACATATATCTAC
AATAGACATCGAGCCGGAAGGTGATGTTTACTTTCCTGAAATCCCCAGCAATTTTAGGCCAGTTTTTAC
CCAAGACTTCGCCTCTAACATAAATTATAGTTACCAAATCTGGCAAAAGGGT

DfrA NNNNN

Ribosomal binding sites
Strong:
AAGGAAATAAGGAGCTGTAGGAT
Weak:
GTATAATCGGCTAGTTCATAGTCGTT

DAM methylation mutations at origin
Original sequence:
AGCGTCAGACCACGTAGAAAA**GATC**AAAG**GATC**TTCTTGA**GATC**CTTTTTTTCTGCGCGTAA
New sequence:
AGCGTCAGACCACGTAGAAAA**GATT**AAAG**GGTC**TTCTTGA**GATC**CTTTTTTTCTGCGCGTAA

Primers for barcode amplification
5’:
AATGATACGGCGACCACCGAGATCTACACNNNNNACACTCTTTCCCTACACGAC
3':

CAAGCAGAAGACGGCATACGAGATNNNNNGTGACTGGAGTTCAGACGTG

Timestamp barcode **NNNNN**
