# Supplementary material for: Intracellular competition shapes plasmid population dynamics
Source: bioRxiv. 2025 Feb 20:2025.02.19.639193. Preprint. [Version 1] doi: 10.1101/2025.02.19.639193 (PMC11870584; doi:10.1101/2025.02.19.639193)
Supplement: 1 [file NIHPP2025.02.19.639193V1-supplement-1.pdf]

bioRxiv preprint doi: https://doi.org/10.1101/2025.02.19.639193; this version posted February 21, 2025. The copyright holder for this preprint (which was not certified by peer review) is the author/funder, who has granted bioRxiv a license to display the preprint in perpetuity. It is made available under aCC-BY 4.0 International license.

Supplementary Figures

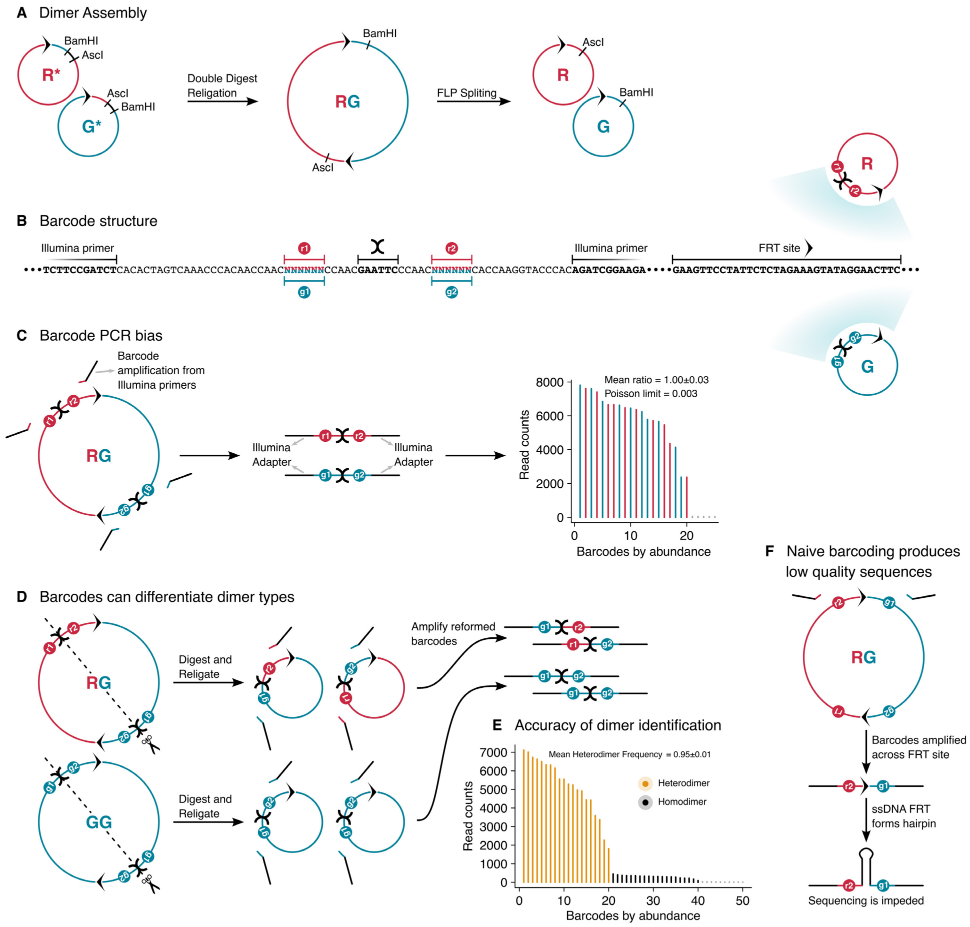

Figure S1: Dimer construction and barcoding

A, Plasmid dimers RG were constructed from arbitrary monomers R and G by adding a pair of restriction sites (BamHI and AscI) at the same position but in reverse order for each monomer. A double digestion and religation creates the dimer, and upon FLP induction the monomers are regenerated within the cell, bearing a restriction scar. **B**, Plasmids were also barcoded. Each monomer received two barcodes separated by an EcoRI site and flanked by Illumina primer sequences that allow for simple downstream barcode sequencing. **C**, Dimers provide an internal control for PCR bias in barcode amplification. We mixed 10 independently barcoded dimers and amplified and sequenced barcode regions from that mix. Ratios between counts of barcodes belonging to the same dimer were on average 1.00 ± 0.03. **D**, To discriminate heterodimers from homodimers, purified dimers were cut at the EcoRI site and religated, uniting barcodes from each side of the dimer. Those reformed barcodes were amplified and sequenced. **E**, Accuracy of dimer discrimination was established by mixing 10 independently barcoded heterodimers and creating religated barcodes from that mix. 95% of reads pointed to heterodimers, displaying a small underestimation of heterodimers. No hybrid barcodes were seen, showing that no cross-ligation between plasmids or PCR-driven hybrids occur. **F**, A naive barcoding system with no digestion and religation fails because of DNA secondary structures during sequencing.

bioRxiv preprint doi: https://doi.org/10.1101/2025.02.19.639193; this version posted February 21, 2025. The copyright holder for this preprint (which was not certified by peer review) is the author/funder, who has granted bioRxiv a license to display the preprint in perpetuity. It is made available under aCC-BY 4.0 International license.

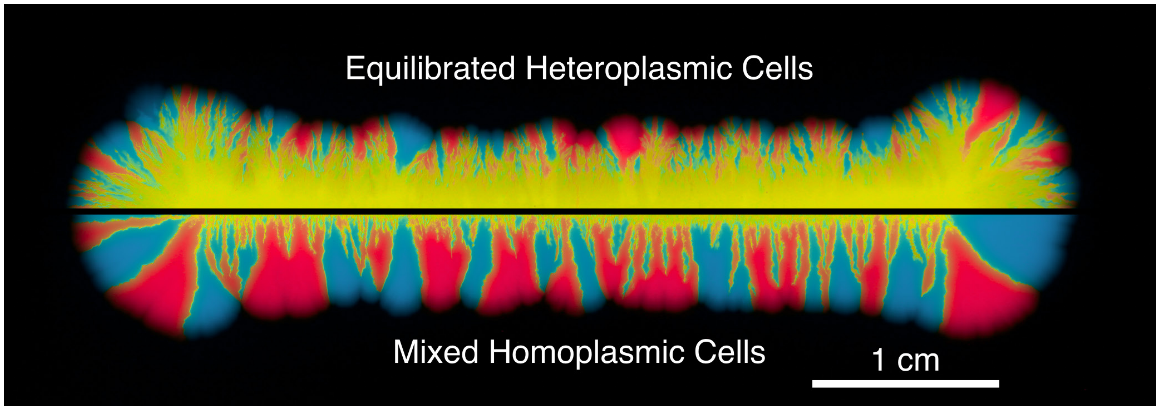

Figure S2: Mixed cells readily segregate as colony expands
Comparison between the rapid segregation of a colony expanding from a linear inoculum containing a mix of homoplasmic cells carrying either pScar or pWater (lower half), versus the much slower segregation that occurs when the inoculum cells carried pScar-pWater dimers, which upon FLP induction led to a population of heteroplasmic cells each carrying an equilibrated amount of pScar and pWater (upper half).

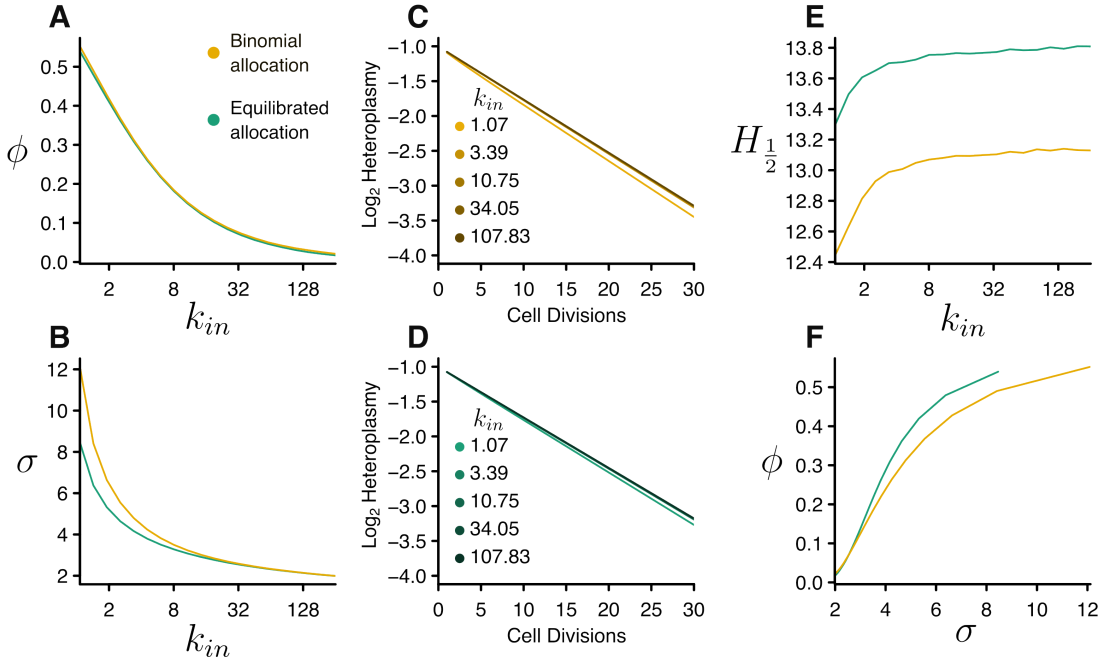

Figure S3: Mechanistic model of plasmid replication imposes strict relationships between parameters
Yellow lines show results obtained for model with binomial plasmid partitioning, and green lines for model with equitable partitioning. **A,B**, Standard deviation σ and autocorrelation ϕ of stationary distributions are shown for each value of k_{in}. Stricter replication control decreases variances and makes them less heritable. **C,D**, Heteroplasmy is shown to decay linearly across conditions. **E**, modeled heteroplasmy decay rate is much faster than seen empirically.**F**, Mechanistic model imposes a strict relationship between and ϕ.

bioRxiv preprint doi: https://doi.org/10.1101/2025.02.19.639193; this version posted February 21, 2025. The copyright holder for this preprint (which was not certified by peer review) is the author/funder, who has granted bioRxiv a license to display the preprint in perpetuity. It is made available under aCC-BY 4.0 International license.

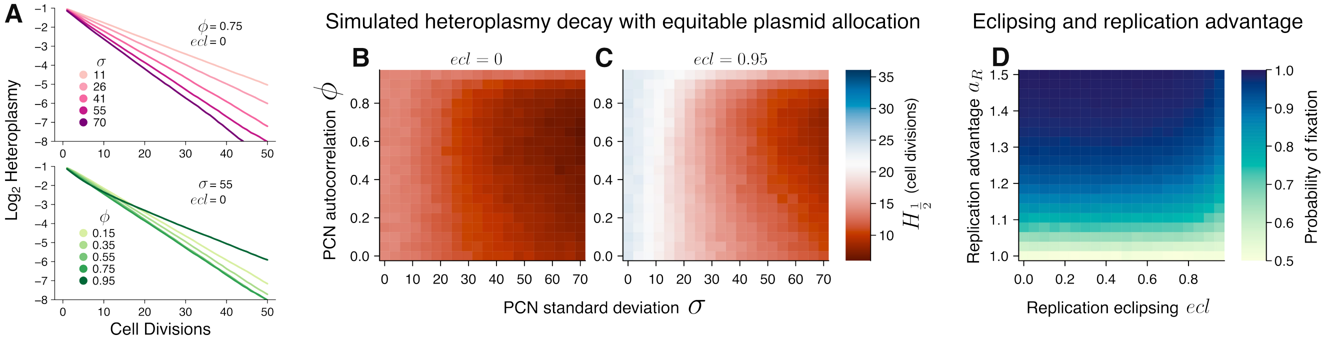

Figure S4: Exponential decay of heteroplasmy

A, Heteroplasmy decay is shown for a series of values of σ and ϕ. Higher σ accelerates heteroplasmy decay. Conversely, increasing ϕ leads to a bi-phasic decay, with an initially fast rate, and a slower asymptotic rate. This bi-phasic behavior occurs because when cells retain similar plasmid copy numbers for many generations there is an initial phase where plasmid fixation quickly occurs across the cell sub-population that has low copy numbers, while co-occurrence is maintained in the subpopulation with high copy numbers. In a slower time scale, cells with many plasmid copies eventually give rise to cells with few copies, and then fixation occurs in those cells, leading to the second, slower fixation phase. **B,C**, Heteroplasmy decay rates $H_{\frac{1}{2}}$ are shown for a modified model in which plasmids are not binomially allocated between daughter cells, but rather each daughter cell receives the same amount of plasmids. This leads to overall slower heteroplasmy decay, but without eclipsing (**B**) still much faster than experimentally observed. **D**, Probability of fixation of a plasmid that has a within-cell competitive advantage is shown as a function of the competitive advantage a_R and the eclipsing factor ecl. Higher advantage increases the fixation probability, but stronger eclipsing can diminish the magnitude of the fixation probability increase.

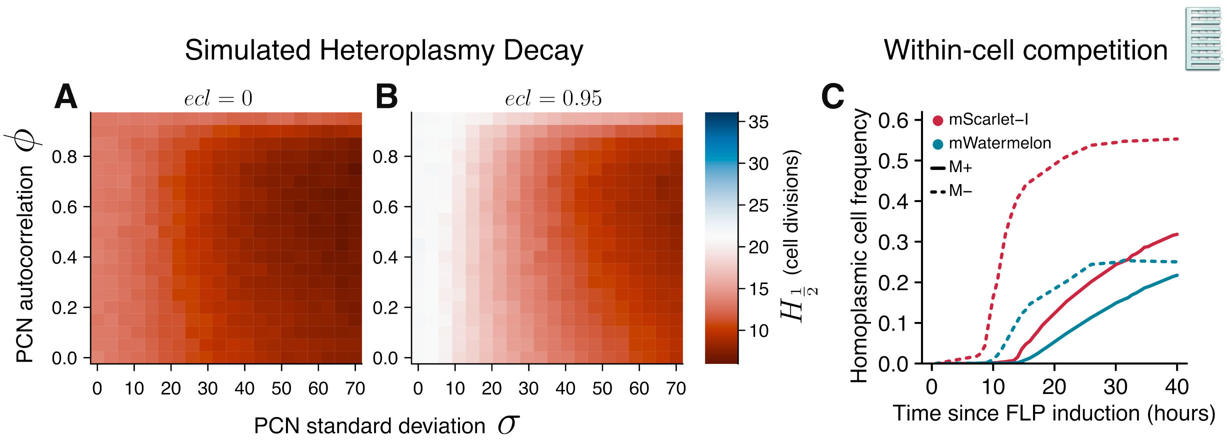

Figure S5: DAM methylation extends within-cell plasmid co-occurrence

A,**B**, Values of $H_{\frac{1}{2}}$ arising from plasmid segregation simulations with (**B**) and without (**A**) eclipsing are shown in red for parameter combinations that lead to faster-than-observed segregation and in blue for slower-than-observed segregation. **C**, Time course of the proportion of cells within a mother machine that fixed one of the competing plasmids. Solid lines represents experiments initiated with cells carrying plasmid dimers with the native methylation sites (pScar-pWater) and dashed lines represent experiments initiated with cells carrying dimers with ablated methylation sites (pScarM^{-}-pWaterM^{-}).

bioRxiv preprint doi: https://doi.org/10.1101/2025.02.19.639193; this version posted February 21, 2025. The copyright holder for this preprint (which was not certified by peer review) is the author/funder, who has granted bioRxiv a license to display the preprint in perpetuity. It is made available under aCC-BY 4.0 International license.

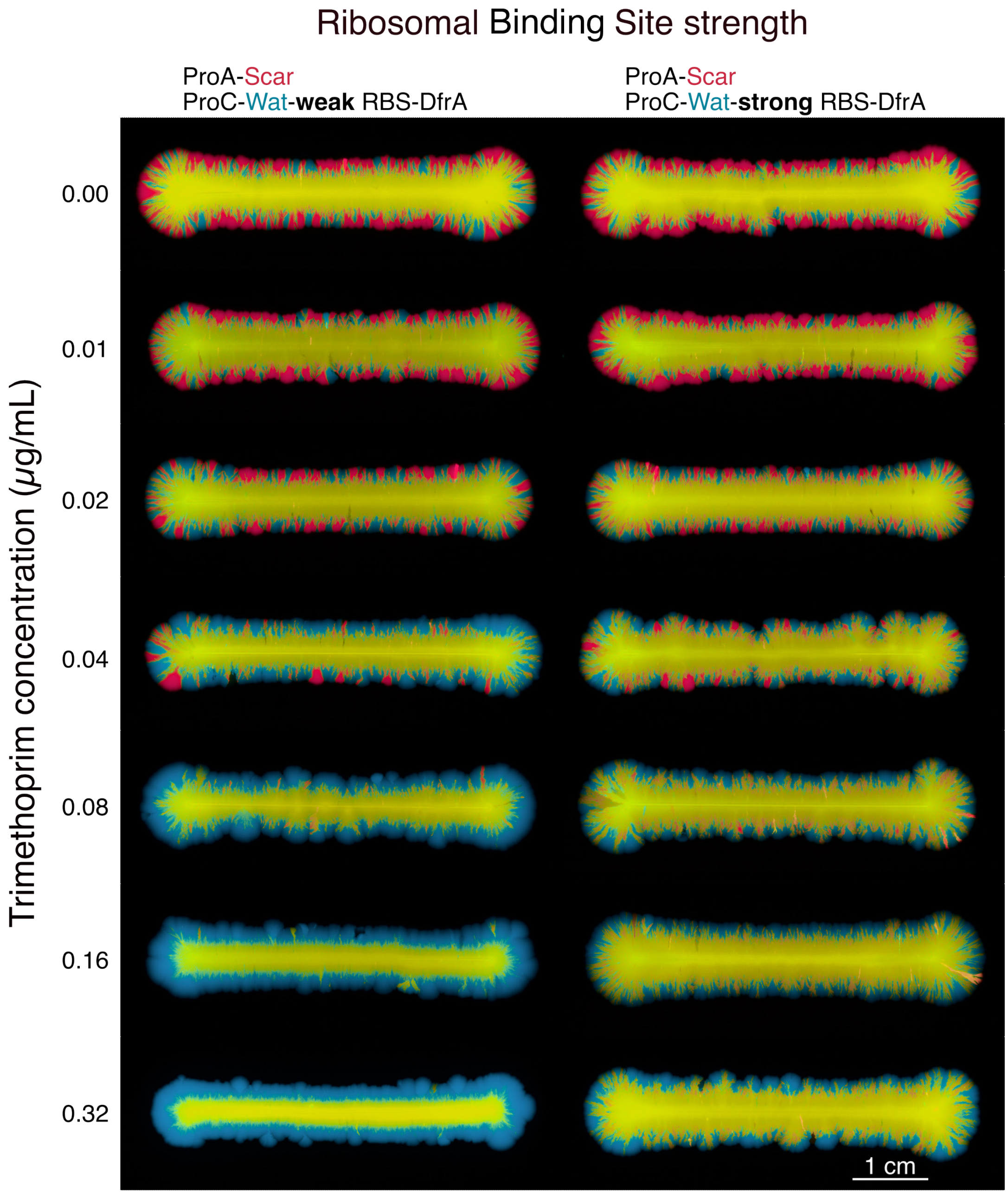

Figure S6: Equilibrated competition experiments
Randomly selected samples of competition experiments under various antibiotic concentrations. Cells were inoculated with a razor blade and carried dimers composed of a non-resistance, low-transcription plasmid (shown in red) and a TMP resistance, high-transcription plasmid (shown in blue). Hue denotes the relative normalized brightness of each fluorescent channel.

bioRxiv preprint doi: https://doi.org/10.1101/2025.02.19.639193; this version posted February 21, 2025. The copyright holder for this preprint (which was not certified by peer review) is the author/funder, who has granted bioRxiv a license to display the preprint in perpetuity. It is made available under aCC-BY 4.0 International license.

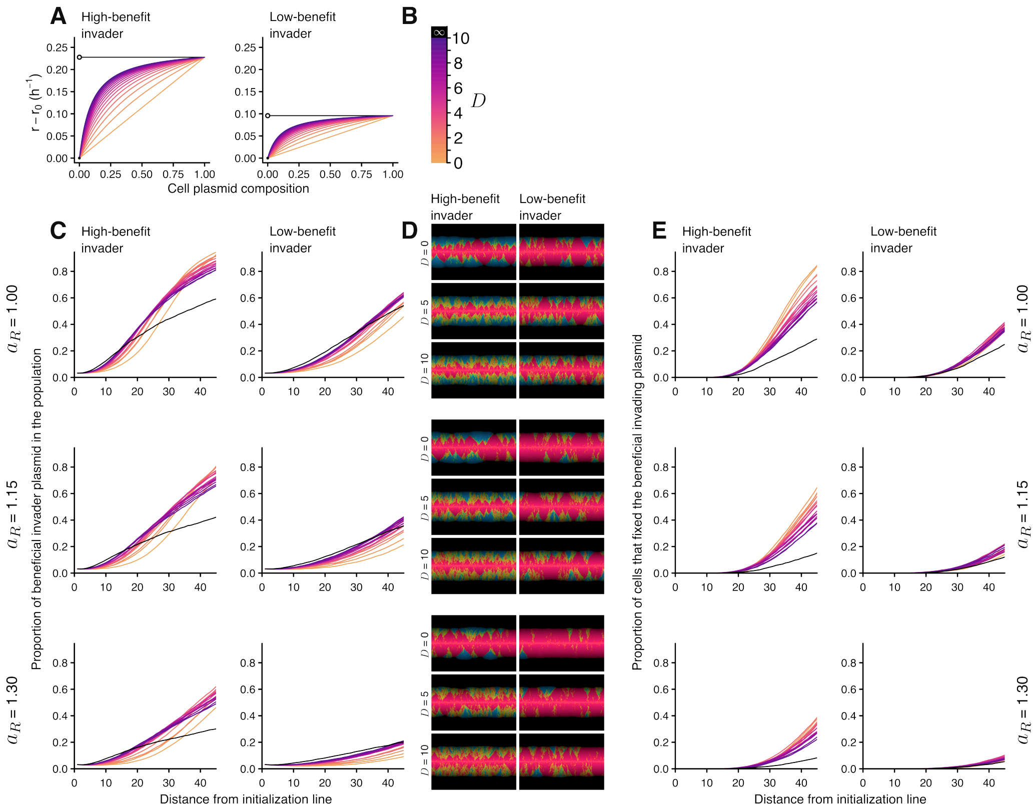

Figure S7: Dominance and within-cell competition at the invasion of a beneficial plasmid
A, Relative fitness as a function of plasmid composition for cells containing a no-benefit plasmid and either a high benefit plasmid or a low-benefit plasmid. Cell fitness profiles are shown for different potential values of dominance D. Black lines show the fitness profile for a completely dominant trait, where any amount of the beneficial plasmid confers the full fitness benefit for the host cell. **B**, Color code for dominance values used throughout the figure. **C**, Average frequency of the invading plasmid as a function of distance from initialization line for high- and low-benefit invading plasmids competing with a no-benefit resident plasmid with a within-cell replication advantage given by a_R. Strong dominance always promotes early frequency gains for the beneficial invader. After the initial boost in invader frequency, higher dominance slows subsequent increases of the high-benefit invader, but intermediate dominance still favors the increases of the low-benefit invader. Overall, under stronger within-cell competition (larger a_R), dominance becomes more favorable for the invader. **D**, Simulation samples. The average frequency of invading plasmids can increase by either progressively increasing across cells (yellow areas), or by the appearance of homoplasmic cells carrying only the beneficial plasmid which then expand as a clonal sector (blue areas). In general, higher dominance favors progressive increase and lower dominance favors expansion of homoplasmic sectors. **E**, Spread of homoplasmic (blue) sectors depends on the rate of appearance of homoplasmic cells and the fitness differential between the homoplasmic cells and their surroundings. Increasing dominance can provide an initial frequency boost for the invading plasmid, increasing the stochastic emergence of homoplasmic cells. However, higher dominance also flattens the fitness differential between emergent homoplasmic cells and their surroundings. For high-benefit plasmids, low dominance already allows for frequent emergence of homoplasmic cells, and, therefore, increased dominance only slows the spread of homoplasmic sectors. Conversely, for low-benefit plasmids, intermediate dominance increases the emergence frequency of homoplasmic cells, and overall favors the increase of such sectors.

bioRxiv preprint doi: https://doi.org/10.1101/2025.02.19.639193; this version posted February 21, 2025. The copyright holder for this preprint (which was not certified by peer review) is the author/funder, who has granted bioRxiv a license to display the preprint in perpetuity. It is made available under aCC-BY 4.0 International license.

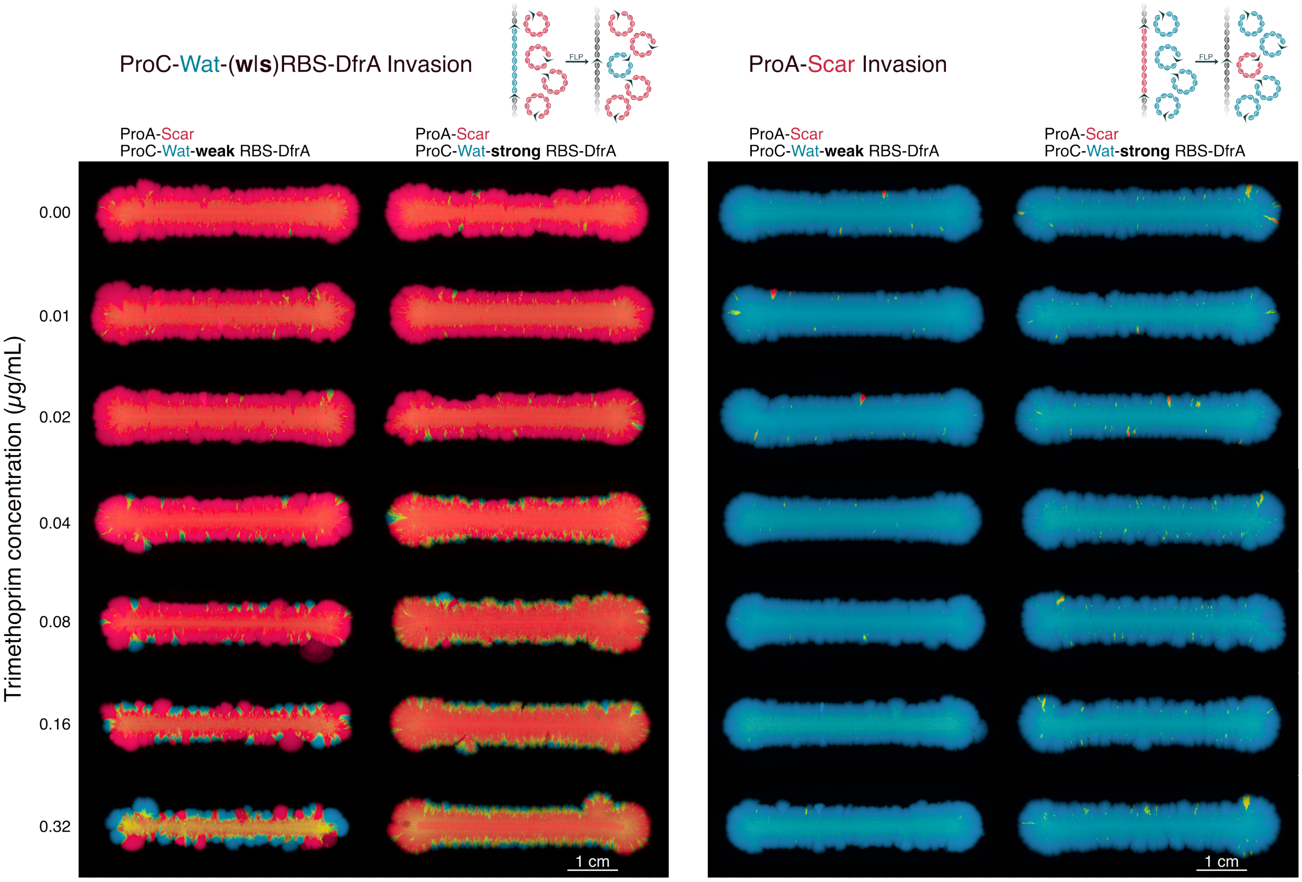

Figure S8: Invasion experiments

Randomly selected samples of invasion experiments under various antibiotic concentrations. Cells were inoculated with a razor blade and carried a resident, free plasmid and another plasmid integrated into the chromosome. Upon FLP-induction, the integrated plasmid was excised and started competing with the resident plasmid, approximating a biological invasion. Left panels show experiments for a resident non-resistance, low-transcription plasmid (shown in red) and a chromosomally integrated TMP resistance, high-transcription plasmid (shown in blue). Right panels show experiments for a resident TMP resistance, high-transcription plasmid (shown in blue) and a chromosomally integrated non-resistance, low-transcription plasmid (shown in red).

bioRxiv preprint doi: https://doi.org/10.1101/2025.02.19.639193; this version posted February 21, 2025. The copyright holder for this preprint (which was not certified by peer review) is the author/funder, who has granted bioRxiv a license to display the preprint in perpetuity. It is made available under aCC-BY 4.0 International license.

Supplemental Movie Captions

Movie S1: pScar-pWater competition
Time lapse of a razor-blade (3.8cm) inoculation of pScar-pWater dimers 12 hours after inoculation, starting immediately after dimer splitting.

Movie S2: Mother machine competition example
Mother machine inoculated with a mixture of cells carrying different dimers. First each lane becomes clonal, and then plasmids are split.

Movie S3: pScar-pWater competition results in the mother machine

Movie S4: Mother machine simulation with mechanistic model
Results are similar to mother machine experiments

Movie S5: ProC_Water(S)DfrA-ProA_scar competition under 0.04µg/mL TMP
Setup is as in Movie S1.
